# Dr. Suhas Vitthal Mapuskar: A Pioneer in Rural Sanitation in Maharashtra, India

**DOI:** 10.7759/cureus.78124

**Published:** 2025-01-28

**Authors:** Umesh Kawalkar, Amrit Mishra, Amar Mankar, K. Mahesh P Rao, Nilesh R Jadhao

**Affiliations:** 1 Community Medicine, Government Medical College, Akola, Akola, IND; 2 Community Medicine, Datta Meghe Institute of Higher Education and Research, Wardha, IND

**Keywords:** community participation, defecation, health promotion, sanitation, toilet facilities

## Abstract

Dr. Suhas Vitthal Mapuskar was an Indian doctor and a social activist who revolutionized rural sanitation practices way before it became a national priority. Nicknamed affectionately as "Dr. Sandas" (Dr. Toilet), he was posthumously awarded the Padma Shri in 2017 for his contributions. At the beginning of his journey in 1959, he identified open defecation as a root cause of health issues and initiated community awareness in Dehu village, Maharashtra. His work involved demonstrating the health risks associated with poor sanitation, conducting deworming campaigns, and developing cost-effective, locally sustainable toilet designs. Dr. Mapuskar emphasized community participation in achieving the target of 90% sanitation coverage. He further promoted the utilization of biogas toilets, which provided hygienic waste disposal and methane gas for fuel, eventually developing the "Malprabha" biogas toilet. Dr. Mapuskar expanded his efforts across Maharashtra through his NGOs. His dedicated efforts have inspired public health professionals and policymakers and exemplify how innovation, community involvement, and appropriate technology can drive toward positive societal change.

## Introduction and background

**Figure 1:**
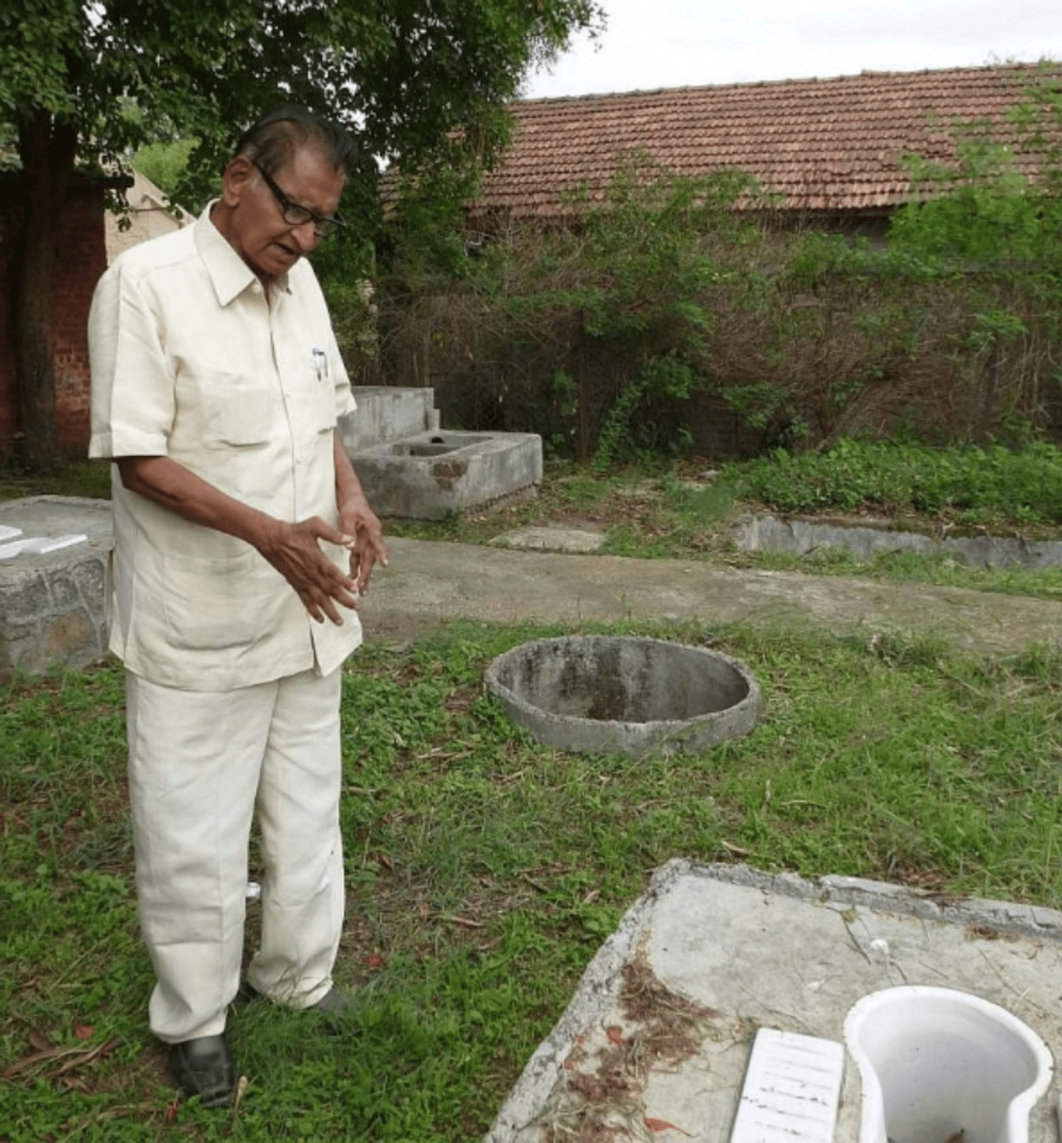
Dr. Suhas Vitthal Mapuskar Under Creative Commons Attribution-Noncommercial-Share Alike 4.0 License. Permission to reproduce this image has been obtained. Credit: Chicu Lokgariwar

Dr. Suhas Vitthal Mapuskar (Figure 1) was a visionary Indian doctor and social activist, who transformed rural sanitation practices long before sanitation became a national priority. Affectionately nicknamed Dr. Sandas (meaning "Dr. Toilet" in Marathi), he received the "Padma Shri," India's fourth highest civilian award, posthumously in 2017 for his contributions. He also received the "Nirmal Gram" award from the former President of India Dr. A.P.J. Abdul Kalam in 2006, for his tireless efforts to eradicate open defecation in Dehu village of Maharashtra since the 1960s [1].

## Review

Vision for sanitation

Dr. Mapuskar's journey toward improving rural sanitation began shortly after he completed his MBBS degree. In 1959, he was posted as the medical officer at the Primary Health Center in Dehu village of Maharashtra. At that time, open defecation was a rampant practice. This led to hygiene-related illnesses and waterborne diseases. Dr. Mapuskar realized this and resolved to tackle the root cause. The First Five-Year Plan launched the sanitation program for the first time in India in 1954 regarding the hygiene and sanitation of rural and urban settings [1].

Initially, Dr. Mapuskar started raising awareness within the community. In 1963, he conducted a worm infestation survey across the village, finding that 86% of the population had worm infestation [2]. He utilized light microscopes to demonstrate the link between infected human feces and contaminated soil to convince the villagers to practice proper sanitation. The burden of worm infestation had been reduced after the intervention of Dr. Mapuskar in that area. The government provided the deworming medication to the entire village, and Dr. Mapuskar stressed to the villagers that this deworming had to be done every three months. Thus began his tireless campaign against open defecation [1].

The development of appropriate technology for rural sanitation

As Dr. Mapuskar explored solutions to the sanitation problem, he came across the WHO guideline on “Excreta disposal for rural areas and small communities” [3]. However, based on the guidelines, the toilets he had constructed collapsed during the monsoon, highlighting the need for locally acceptable and suitable designs. This failure motivated him to look for "appropriate technology" toilet designs and develop sustainable and cost-effective toilet facilities for the village [1].

Community participation

Dr. Mapuskar believed that any new initiative's success depends on the community's involvement and participation in both the decision-making and implementation processes. He worked meticulously with the village Gram Sabha to secure the funding for the sanitation project. He further encouraged the development of a committee to supervise the construction of toilets. This also proved to be useful for women and children as they didn't have to go out in the dark for open defecation. This sense of community participation played a crucial role in the success of the sanitation campaign in Dehu. The villagers built over 100 toilets monthly on a "no profit and no loss" basis. By 1980, the village had achieved 90% coverage, an astounding milestone in rural India [1].

In 1980, Dr. Mapuskar promoted a biogas toilet design developed by Shri Sitaram Purushottam Patwardhan (a.k.a. Appasaheb Patwardhan and "Gandhi of Konkan"). This innovative technology not only provided a hygienic waste disposal solution but also generated methane gas, which could be used as a fuel [4]. It addressed both the sanitation and the energy efficiency issues prevalent in villages. The first of these biogas toilets was installed by a resident, Mrs. Pardeshi, who realized its income-generation potential. For five years, Dr. Mapuskar modified the biogas toilet design and developed the "Malprabha" biogas toilet [5].

The big picture from Dehu to Maharashtra

After Dehu, Dr. Mapuskar expanded his efforts across Maharashtra through the establishment of two non-governmental organizations (NGOs), namely "Appasaheb Patwardhan Safari Wa Paryawaran Tantraniketan" and "Jyotsna Aarogya Prabodhan," to work on health awareness and appropriate technology. Another of his groundbreaking initiatives was the Decentralised On-Site Integrated Waste Management (DOSIWAM) system, combining sanitation with solid waste and vegetable waste management to promote health and sustainability. This model was adopted at over 25 sites across India and has also been adopted by the government, as similar problems were faced all over the country [5].

Recognition and legacy

Dr. Suhas Mapuskar's legacy lives on through the healthcare models he helped develop and the many professionals he inspired. His work continues to influence public health strategies, especially in rural and resource-limited settings. His emphasis on community involvement and sustainable healthcare solutions remains relevant in contemporary public health discourse. His daughter, Dr. Shilpa Narayanan, carries forward his mission through their NGO. They have impacted thousands of lives by fostering a culture that hygiene and sanitation are a community responsibility [5].

## Conclusions

Dr. Suhas Vitthal Mapuskar’s work in transforming rural sanitation stands as an example of the power of innovation, community participation, and appropriate technology. His diligent efforts in Dehu village and later across the state of Maharashtra have inspired a generation of public health experts and policymakers. His story is a reminder of how one person’s vision and perseverance can bring about a positive change in society.
